# Behavioral Variables to Assess the Toxicity of Unionized Ammonia in Aquatic Snails: Integrating Movement and Feeding Parameters

**DOI:** 10.1007/s00244-022-00920-z

**Published:** 2022-03-24

**Authors:** Álvaro Alonso, Gloria Gómez-de-Prado, Alberto Romero-Blanco

**Affiliations:** Universidad de Alcalá, Facultad de Ciencias, Departamento de Ciencias de la Vida, Unidad Docente de Ecología, Biological Invasions Research Group, Universidad de Alcalá, Plaza de San Diego S/N, 28801 Alcalá de Henares, Madrid Spain

## Abstract

Behavioral endpoints are important parameters to assess the effects of toxicants on aquatic animals. These endpoints are useful in ecotoxicology because several toxicants modify the animal behavior, which may cause adverse effects at higher levels of ecological organization. However, for the development of new bioassays and for including the behavior in ecotoxicological risk assessment, the comparison of sensitivity between different behavioral endpoints is necessary. Additionally, some toxicants remain in aquatic environments for a few hours or days, which may lead to animal recovery after toxicant exposure. Our study aimed to assess the effect of unionized ammonia on the movement and feeding behaviors of the aquatic gastropod *Potamopyrgus antipodarum* (Tateidae, Mollusca) and its recovery after exposure. Four treatments were used: a control and three nominal concentrations of unionized ammonia (0.25, 0.5 and 1 mg N-NH_3_/L). Each treatment was replicated eight times, with six animals in each replicate. Animals were exposed to unionized ammonia for 48 h (exposure period) and, subsequently, to control water for 144 h (post-exposure period). Two movement variables were monitored without food and five feeding behavioral variables were monitored in the presence of food. Some of the feeding behavioral variables showed higher sensitivity (LOEC = 0.25–0.5 mg N-NH_3_/L) than the movement behavior variables monitored without food (LOEC = 1 mg N-NH_3_/L). After exposure to unionized ammonia, animals showed a recovery of most behavioral endpoints. The inclusion of post-exposure period and feeding behaviors in bioassays may make studies more realistic, which is crucial for a proper ecotoxicological risk assessment.

**Supplementary Information:**

The online version contains supplementary material available at 10.1007/s00244-022-00920-z.

Behavioral parameters are important variables to assess the deleterious effects of toxicants on several aquatic animals, such as mollusks, decapods, amphipods, annelids, cnidarians, fish (Alonso et al. 2009, 2016; Faimali et al. 2017; Hellou 2011; Melvin and Wilson 2013; Suski et al. 2019). Behavior allows animals to face potential environmental hazards, such as the presence of predators, variation in water chemical properties, increase in competition, or exposure to toxicants (Alonso et al. 2016; Dell’Olmo 2002; Hellou 2011; Little and Finger 1990; Suski et al. 2019). These environmental changes can modify the survival, growth, and reproduction of populations, which can trigger effects at higher levels of ecological organization (Hellou 2011). Thus, the number of behavioral studies to assess effects of toxicants to aquatic animals has increased in the last decades (Melvin and Wilson 2013). Impedance conversion technique, feeding behavior, spatial analysis of behavior, and other techniques have been developed in the last decades for different animal groups, such as mollusks, amphipods, fish (Alonso et al. 2009, 2016; Alonso and Valle-Torres 2018; Delcourt et al. 2013; Dell’Olmo 2002; Faimali et al. 2017; Kirkpatrick et al. 2006; Nørum et al. 2011). In general, bioassays based on behavior are less time-consuming than developmental and reproduction bioassays, and they present a higher sensitivity than lethal bioassays (Melvin and Wilson 2013). However, there are multiple behaviors that can be monitored, such as movements, feeding, or avoidance among others (Alonso and Camargo 2009a; Alonso and Valle-Torres 2018; Araujo and Blasco 2019; Dell’Olmo 2002; Kirkpatrick et al. 2006). These behaviors have been tested for different invertebrate and vertebrate animals (Alonso and Valle-Torres 2018; Bownik et al. 2020; Dell’Olmo 2002; Hellou 2011). Assessing the sensitivities to toxicants of those behaviors is important to develop new behavioral bioassays and to include specific behaviors in the ecotoxicological risk assessment of toxicants.

Animal movements, such as swimming, avoidance, sliding, or time to start activity, allow animals avoiding predation, escaping from polluted areas, locating food, or reproducing. Therefore, assessing the effects of toxicants on movements may help make laboratory bioassays more realistic. Among behaviors, feeding behaviors (i.e., any animal action allowing it to obtain food) have an essential ecological relevance: any toxicant that reduces the capacity of animals to locate, reach food, or feed may imply effects at higher levels of ecological organization (Alonso et al. 2009, 2016; Alonso and Valle-Torres 2018; Couland et al. 2015; Maltby et al. 1990; Nyman et al. 2013). Those effects are primarily caused by the reduction in the energy intake, which subsequently produces deleterious effects on growth and reproduction (Couland et al. 2015; Maltby et al. 1990). Animal movements and feeding behaviors are closely related, as movements allow animals to reach the food and feed. However, in behavioral bioassays the assessment of movement is usually conducted in absence of food (Alonso and Camargo 2009a; Bownik et al. 2020; Liu et al. 2020). Additionally, food releases chemical cues which mediate behaviors such as movements or food selection (Costa and Nomura 2014; Hassenkloever et al. 2012; Lahman and Moore 2015; Martin 2017; Alonso 2021). In that case, the locomotory system is involved in the behavioral reaction, but also the olfactory system which is essential for the orientation abilities of aquatic animals (Azizishrazi et al. 2013; Lahman and Moore 2015; Mahabir and Gerlai 2017).

The assessment of the degree of behavioral recovery after toxicant exposure is a relevant issue in ecotoxicological studies (Alonso and Camargo 2014; Cao et al. 2014; Lahman and Moore 2015). In some pollution events, toxicants are released to the aquatic media for a few hours or days, causing an adverse effect on behavior. Subsequently, behavior of pre-exposure animals may recover completely or the adverse effects may increase. Recovery depends on the kind of toxicant, its concentration, and exposure time (Alonso and Camargo 2009b, 2014; Gordon et al. 2012; Hoang et al. 2007; Lahman and Moore 2015). Therefore, the inclusion of recovery after exposure to toxicants in the experimental setup is a relevant issue to improve the extrapolation of results from laboratory bioassays to natural ecosystems.

Among toxicants, ammonia has previously shown deleterious effects on different behavioral parameters (Alonso and Camargo 2004, 2009a; Beggel et al. 2017; Richardson et al. 2001). Different human activities (e.g., farming runoff, atmospheric deposition, industrial wastes, and urban effluents) have increased the ammonia concentration in aquatic ecosystems (Camargo and Alonso 2006; Constable et al. 2003; Vitousek et al. 1997; Zhang et al. 2017, 2018). In fact, ammonia is one of the most widespread contaminants in developed countries (Abel 2000; Spencer et al. 2008). Ammonia is an inorganic compound that comes mainly from the decomposition of organic matter, with an equilibrium between the ionized (NH_4_^+^) and the unionized (NH_3_) forms (Constable et al. 2003). The proportion of each form in freshwater ecosystems is mainly controlled by pH and water temperature (Emerson et al. 1975), with the unionized form being the most toxic (Camargo and Alonso 2006; Constable et al. 2003).

The aim of this study was to test the effect of unionized ammonia on the movement and feeding behaviors of the aquatic gastropod *Potamopyrgus antipodarum* (Tateidae, Mollusca), including the degree of behavioral recovery after toxicant exposure. This species has been broadly used in behavioral studies, mainly due to its sensitivity and easy cultivation in laboratory (Alonso and Camargo 2009a; Alonso, García-Perinan and Camargo 2016; Alonso and Valle-Torres 2018; Heye et al. 2019; Ruppert et al. 2016).

## Materials and Methods

### Animal Culture

Animals for the bioassay were obtained from our culture at the Laboratory of Ecology (Department of Life Sciences, University of Alcalá). The culture of *P. antipodarum* was started in 2009, with animals collected in the upper reach of the Henares River (Guadalajara province, Spain). Culture tanks consisted of 60L of standardized USEPA water (96 mg NaHCO_3_, 60 mg CaSO_4_·2H_2_O, 4 mg KCl, 122.2 mg MgSO_4_, per liter of deionized water plus 10 mg CaCO_3_ per liter) (USEPA 2002) at 20-22ºC. Snails were fed with 0.10 mg of dry food per animal and day (50% fish food Tetra- Menü© GmbH, Melle, Germany + 50% Sera © Spirulina Tabs GmbH, Heinsberg, Germany). Ten percent of the culture water was renewed every two weeks. Water was filtered by means of aquarium filters.

### Bioassay Design

A behavioral bioassay was conducted using four treatments: a control and three nominal concentrations of unionized ammonia (0.25, 0.5, and 1 mg N-NH_3_/L). These concentrations were used because they were in the range of short-term toxic effects on this species (Alonso and Camargo 2014). Ammonia treatments were prepared from an ammonia stock solution (NH_4_Cl, 99.5% purity, Panreac, Spain). Water temperature and pH were monitored during the bioassay to estimate the unionized ammonia concentration (Emerson et al. 1975). The control and each treatment were replicated eight times, with six animals in each replicate. Therefore, each treatment (including the control) was conducted with 8 replicates and 48 animals (6 animals in each replicate). The total number of animals for the bioassay was 192 animals in 32 replicates (8 replicates in each treatment including control). Animals were exposed to unionized ammonia for 48 h (exposure period with one monitoring time at 48 h) and, subsequently, transferred to control water for 144 h (post-exposure period with two monitoring times at 48 and 144 h). The total duration of the bioassay (including exposure and post-exposure) was 8 days. The bioassay was conducted at 18ºC in a climatic chamber (ANSONIC®VAC0732). The previous week to the start of the bioassay, 200 animals were kept in four vessels (700 ml) in the climatic chamber at 18ºC for acclimatization. During this period, animals were feed ad libitum with food pellets (JBL® NovoOrawn GmbH & Co KG, Germany). Total ammonia (NH_3_ plus NH_4_^+^) was analyzed at 0, 24, and 48 h of exposure through a standardized spectrophotometric method (Spectroquant®Merck, Germany, Detection limit = 0.002 mg N-NH_3_/L) (American Public Health Association 2005). Glass vessels of 125 ml of solution were used for each replicate. Each vessel was covered with a perforated Petri dish to reduce ammonia and water evaporation. The experimental setup allowed the assessment of the behavior recovery after the exposure to unionized ammonia.

### Monitored Variables

Two movement variables of *P. antipodarum* were monitored in absence of food: (1) Immobility: an animal was considered immobile when no displacement was observed after 600 s of activity recording, its soft body was inside the shell, and its operculum moved after being touched with forceps (see below). If the operculum did not move, the animal was considered dead. (2) Activity: it was measured as the time (in seconds) taken by each animal to start the sliding movement (Alonso and Camargo 2009a). To monitor this parameter, each animal was taken up with forceps and placed in the center of the bioassay vessel with the operculum facing to the bottom. The time to start the sliding movement was recorded through a chronometer. Both variables were monitored at 48 h of unionized ammonia exposure in testing water and at 48 and 144 h of post-exposure in control water. These variables were monitored in absence of food using a stereomicroscope (MOTIC® SMZ-168) with a fiber optic light (Jenalux® 150). With this procedure, we ensured that movements were not affected by the presence of food.

Feeding behaviors were monitored through a video-recording method (Alonso et al. 2016). Eight Petri dishes (130 mm diameter and 75 ml of USEPA water) were placed on a table. A video camera (Canon LEGRIA HF R57) was placed 104 cm above the dishes with a tripod. In each dish, six animals of a replicate were situated. A total of 4 batches were recorded. In each batch, two replicates of each treatment were monitored (e.g., 4 treatments X 2 replicates = 8 dishes with 6 animals in each dish, 48 animals monitored in each batch). Eight food pellets (JBL® NovoOrawn GmbH & Co KG, Germany) were placed in one extreme of each dish and the six animals of a replicate in the opposite side of the dish. For the video-recording and in each batch, one animal of a treatment and replicate was placed in its corresponding dish. Subsequently, another animal of the next treatment and replicate was placed in the next dish and so on until all animals of each batch (48 animals) were in all the dishes. For each batch, the video-recording started when the first animal was placed in the first dish and extended for 90 min. Therefore, all replicates in all treatments (including the control) were recorded for 90 min. Videos were analyzed by means of the free software ImageJ 1.52 (Wayne Rasband, National Institute of Health, USA; http://rsb.info.nih.gov/ij/). Five feeding variables were monitored in presence of food: 1) the time taken by the first (t-1), 2) second (t-2), and 3) third (t-3) animal to reach the food pellets. For recording that, the time (in seconds) taken by the first (and second and third animal) to reach the pellets and to contact with them for at least 5 s was directly monitored in videos. 4) The percentage of animals that were eating (% eating). For that, all videos were checked to monitor the number of animals that were over the food or in contact with pellets in each replicate for at least 5 s. 5) The mean distance (in mm) of animals to the food pellets was assessed in each replicate from nine images taken at 10-min intervals. Distances were estimated through image analysis in ImageJ, and the mean of all distances for each replicate was calculated. These five variables were monitored in presence of food at 48 h of exposure and at 48 and 144 h of post-exposure.

At 24 and 48 h of exposure and at 119 h of post-exposure, the dissolved oxygen in water, pH, conductivity, and water temperature were monitored through an oximeter (Crison model oxi 45 +), pH meter (Crison micropH 2001, ALELLA 08,328), and conductivimeter (Crison CM 35 + for conductivity and water temperature). Additionally, the shell length of snails was measured at the end of the bioassay through an ocular micrometer. The mean (± SD) (*n* = 64) shell length of experimental animals was 3.65 ± 0.17 mm (for control, 3.67 ± 0.23 mm, 3.66 ± 0.16 mm for the lowest ammonia concentration, 3.65 ± 0.11 mm for the intermediate concentration, and 3.65 ± 0.18 mm for the highest concentration).

### Statistical Analysis

Differences in mortality between treatments (including control) were assessed by means of a Kruskal–Wallis test. A mixed ANOVA test was applied to analyze the influence of time (48 h of exposure, 48 h and 144 h of post-exposure), treatments (control and three unionized ammonia concentrations), and their interactions (time X treatment) on the movement (immobility and activity) and feeding variables (time taken by the first, second, and third animal to reach the food pellets, the percentage of animals that were eating, and the mean distance (in mm) of animals to the food pellets). If significant effects were obtained for treatment or time (*p* < 0.05), a post hoc test was performed to analyze which treatments caused differences regarding controls or which time of post-exposure caused differences regarding exposure period (Wilcoxon signed rank test with Holm’s correction and pairwise Wilcoxon rank sum tests with Holm’s correction, respectively). For each variable, the highest unionized ammonia concentration not significant different from the control was considered as the No Observed Effect Concentration (NOEC) and the lowest concentration significantly different from the control was considered as the Lowest Observed Effect Concentration (LOEC). We applied the Greenhouse–Geisser approach to the immobility analysis since the sphericity assumption was not fulfilled. Activity, mean distance to the food, and time taken by the first, second, and third animals to reach the food were log-transformed to achieve normality and homogeneity of variances after checking parametric requirements through Kolmogorov–Smirnov (normality) and Fligner–Killeen tests (homocedasticity). We performed all the statistical analyses with R software (R Core Team 2020). For all statistical analyses, dead animals were not considered.

## Results

The mean values (± SD) (*n* = 4–8) of the physical–chemical parameters for the control and 0.25, 0.5, and 1 mg N-NH_3_/L treatments were 6.2 ± 0.26, 6.0 ± 0.26, 6.3 ± 0.17 and 6.3 ± 0.25 mg O_2_/l for dissolved oxygen, 8.0 ± 0.05, 8.1 ± 0.03, 8.0 ± 0.04 and 8.0 ± 0.03 for pH, 367.3 ± 11.9, 364.5 ± 5.92, 368.2 ± 11.3 and 361.8 ± 11.3 µS/cm for conductivity, and 17.7 ± 0.55, 17.9 ± 0.41, 17.8 ± 0.51, and 17.5 ± 0.69 °C for water temperature. The mean (± SD) (*n* = 24) actual concentrations of unionized ammonia were < 0.03 (Control), 0.23 ± 0.03, 0.62 ± 0.14, and 1.5 ± 0.52 mg N-NH_3_/L. We only recorded dead animals in the highest ammonia concentration (4.1 ± 11; mean (*n* = 8) ± SD percentage of mortality at the end of the bioassay), although there were no significant differences of mortality across treatments (*p* = 0.39; Kruskal–Wallis test).

**Fig. 1:**
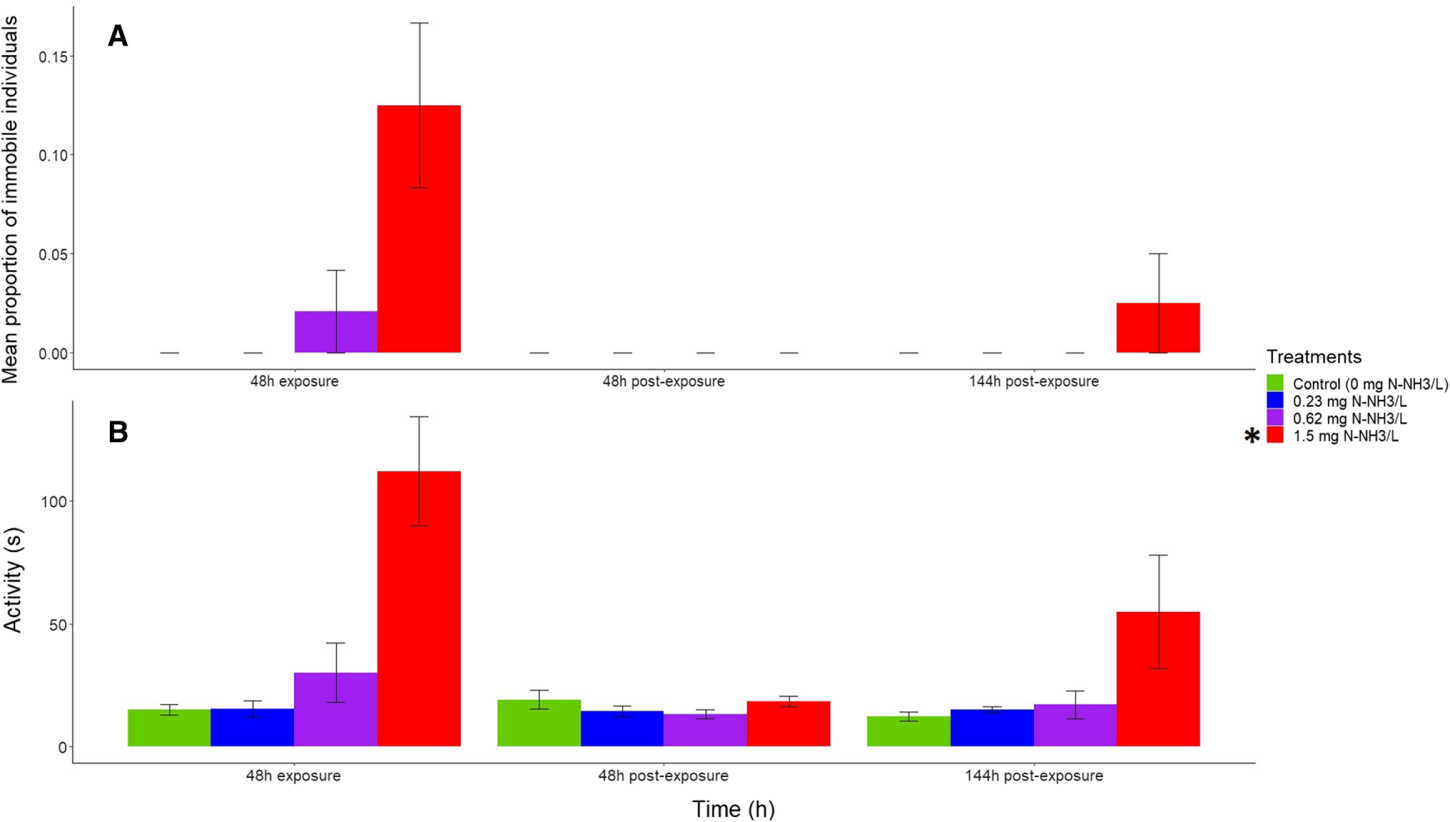
Mean (± SE) proportion of immobile individuals (**A**) and mean (± SE) reaction time (in seconds; **B**) of *Potamopyrgus antipodarum* individuals after 48 h of exposure to ammonia and 48 and 144 h of post-exposure in each treatment (control and three increasing actual unionized ammonia concentrations in mg N-NH_3_/L). The asterisk indicates the ammonia treatment that caused significant differences of the variable with respect to the control across all time exposures and post-exposures (*p* < 0.05, Wilcoxon Signed Rank Test with Holm’s correction). For immobility, 48 h of post-exposure showed differences with 48 h exposure (*p* < 0.05, pairwise Wilcoxon rank sum tests with Holm’s correction). For activity, no significant differences were found between 48 and 144 h of post-exposure and 48 h of exposure (*p* > 0.05, pairwise Wilcoxon rank sum tests with Holm’s correction)

The time, treatment, and their interaction affected movement variables (Table 1; *p* < 0.05, mixed ANOVA). Only the highest concentration of unionized ammonia increased both the proportion of immobile animals and the time taken by each animal to start the sliding movement (Fig. 1 and Table 1) (*p* < 0.05, Wilcoxon signed rank test with Holm’s correction). Therefore, for both variables, the NOEC was 0.62 mg N-NH_3_/L and the LOEC 1.5 mg N-NH_3_/L. In general, responses to the treatments varied with time, leading a significant interaction between both factors (Fig. 1). This was especially marked for the proportion of immobile animals at the highest unionized ammonia concentration, which showed a huge response during the exposure to ammonia, a full recovery at 48 h of post-exposure, and a slight increase at 144 h of post-exposure, whereas responses to remaining treatments were relatively homogeneous across time (Fig. 1A).

**Table 1:** Summary of results of the mixed ANOVA assessing the influence of unionized ammonia treatments, time, and their interaction on the immobility and activity of *Potamopyrgus antipodarum*. The time (48 h of exposure and 48 and 144 h of post-exposure) was the within-subject factor, the treatment (Control, 0.23, 0.62, and 1.5 mg N-NH_3_/L) was the between-subjects factor, and the immobility (percentage of immobile individuals) and activity (in seconds) were the response variables

Source of variation	Degrees of freedom	F	*p*	Effect size
*Immobility*				
Within-subject				
Time	1.2/33.5^a^	5.6	< 0.05	0.1
Time ✕ Treatment	3.6/33.5^a^	3.6	< 0.05	0.13
Between subjects				
Treatment	3/28	11	< 0.0001	0.2
*Activity*				
Within-subject				
time	2/56	6.6	< 0.01	0.13
Time ✕ Treatment	6/56	3.9	< 0.01	0.21
Between subjects				
Treatment	3/28	12.2	< 0.0001	0.32

^a^Degrees of freedom (degrees of freedom of numerator/degrees of freedom of denominator) have been corrected for sphericity using the Greenhouse–Geisser approach (Field et al. 2012)

**Fig. 2:**
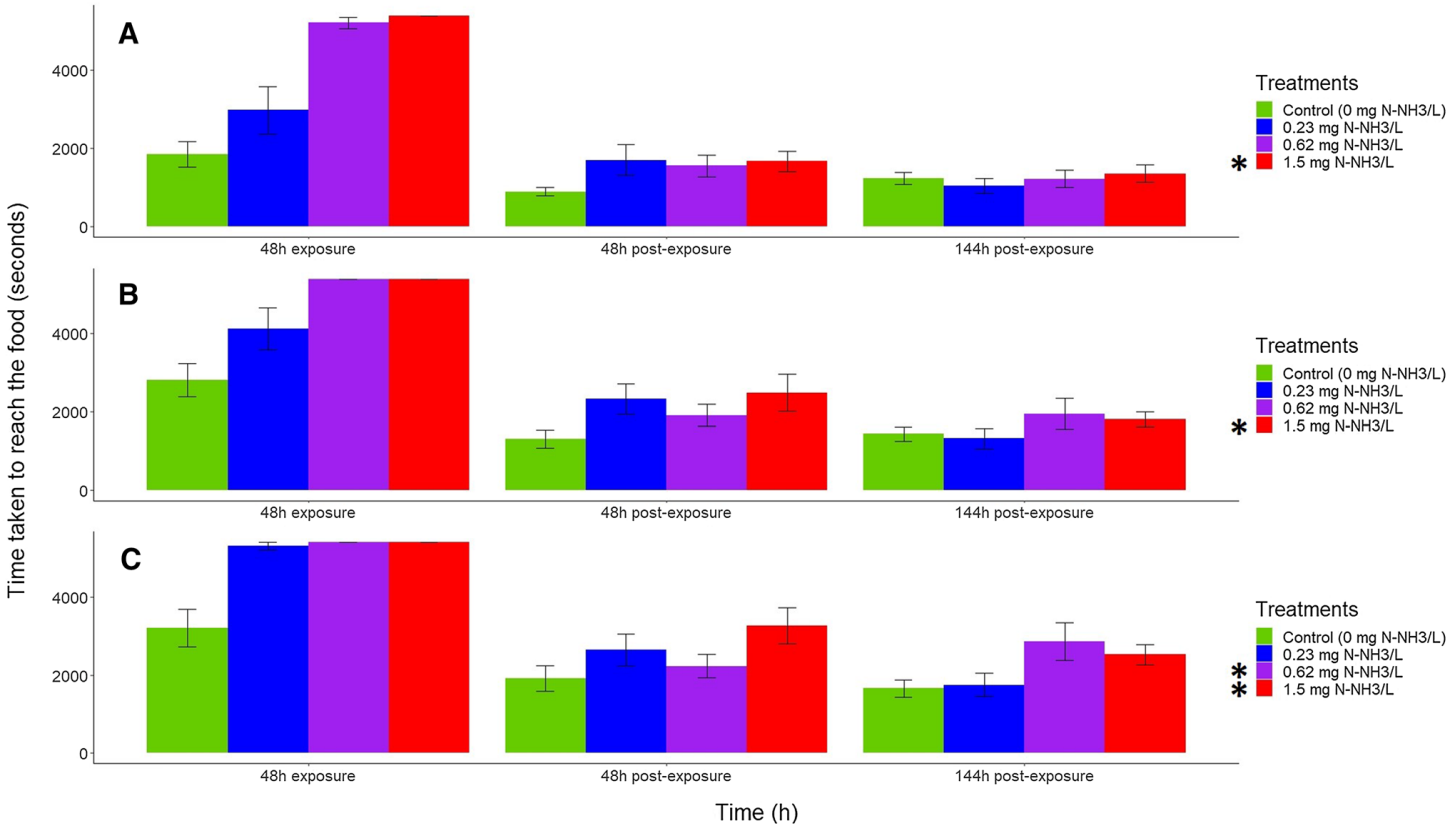
Mean (± SE) time (in seconds) taken by the first (**A**), the second (**B**), and the third (**C**) animals to reach the food pellets after 48 h of exposure to the ammonia treatment and 48 and 144 h of post-exposure (control and three increasing actual unionized ammonia concentrations in mg N-NH_3_/L). The asterisk indicates the ammonia treatment that caused significant differences of the variable with respect to the control across all time exposures and post-exposures (*p* < 0.05, Wilcoxon signed rank test with Holm’s correction). For all variables, responses at 48 and 144 h of post-exposure differed from those at 48 h of exposure (*p* < 0.05, pairwise Wilcoxon rank sum tests with Holm’s correction)

**Fig. 3:**
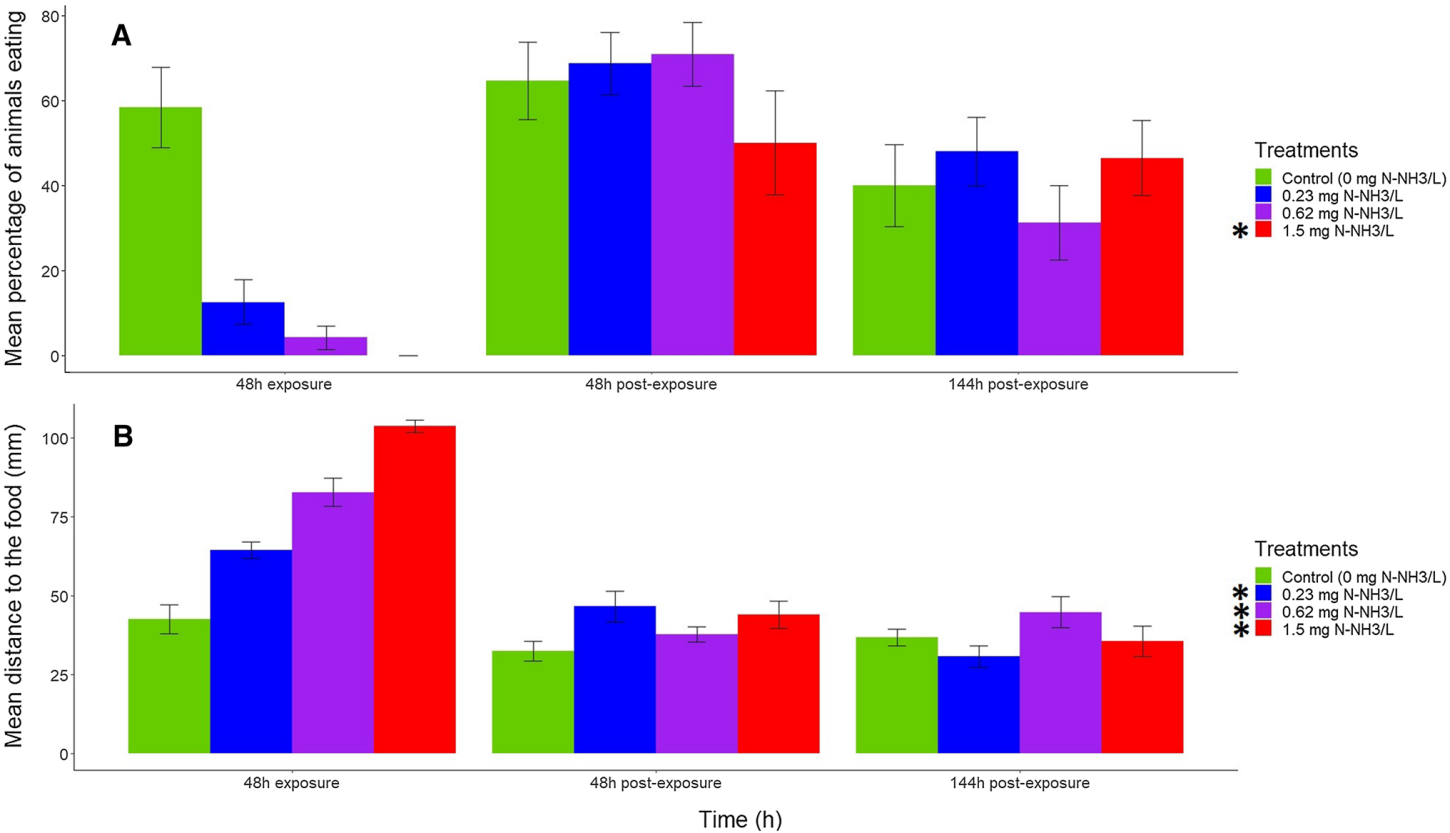
Mean percentage (± SE) of animals that were eating (**A**) and the mean (± SE) distance of animals to the food pellets (in mm) (**B**) for 48 h of exposure and 48 and 144 h of post-exposure (control and three increasing actual unionized ammonia concentrations in mg N-NH_3_/L). Asterisks indicate the ammonia treatments that significantly differed from the control for each variable (*p* < 0.05, Mann–Whitney U test with Holm’s method correction). For all variables, responses at 48 and 144 h of post-exposure different from those at 48 h of exposure (*p* < 0.05, pairwise Wilcoxon rank sum tests with Holm’s correction)

The treatment and time were significant for all feeding behavior variables (Table 2) (*p* < 0.05, mixed ANOVA). Their interaction was also significant for the time taken by the first animal to reach the food, percentage of animals eating, and mean distance to the food (Table 2) (*p* < 0.05, mixed ANOVA). The monitoring of feeding variables showed that the time taken by the first and second animal to reach the food pellets were increased by the highest unionized ammonia concentration with respect to control (Fig. 2) (*p* < 0.05, Wilcoxon signed rank test with Holm’s correction). Therefore, for both variables the NOEC was 0.62 mg N-NH_3_/L and the LOEC 1.5 mg N-NH_3_/L. The time taken by the third animal to reach the food was increased by the two highest unionized ammonia concentrations compared with the control (Fig. 2C) (*p* < 0.05, Wilcoxon signed rank test with Holm’s correction). For this variable, NOEC was 0.23 mg N-NH_3_/L and LOEC was 0.62 mg N-NH_3_/L. The percentage of animals that were eating was only reduced by the highest unionized ammonia concentration (Fig. 3A) (*p* < 0.05, Wilcoxon signed rank test with Holm’s correction) (NOEC = 0.62 mg N-NH_3_/L and LOEC = 1.5 mg N-NH_3_/L). The mean distance of animals to the food pellets was the most sensitive variable, given that it was increased by all unionized ammonia treatments (Fig. 3B) (*p* < 0.05, Wilcoxon signed rank test with Holm’s correction). Therefore, the LOEC was 0.23 mg N-NH_3_/L for this variable. In general, during the exposure period to unionized ammonia, all studied variables responded to the toxicant, but during the post-exposure period, the differences between treatments and control were reduced (Figs. 1, 2, and 3). The percentage of immobile animals was different between 48 h of post-exposure and 48 h of exposure (Fig. 1A) (*p* < 0.05, pairwise Wilcoxon rank sum tests with Holm’s correction). No differences were found for activity between post-exposure times and exposure time (Fig. 1B) (*p* > 0.05, pairwise Wilcoxon rank sum tests with Holm’s correction). All feeding variables differed between 48 and 144 h of post-exposure and 48 h of exposure (Fig. 2 and 3) (*p* < 0.05, pairwise Wilcoxon rank sum tests with Holm’s correction).

**Table 2:** Summary of results of the mixed ANOVA assessing the influence of unionized ammonia treatments, time, and its interaction on the time taken by the first, second, and third animals to reach the food, the percentage of animals eating, and the mean distance to the food of *Potamopyrgus antipodarum*. The time (48 h of exposure and 48 and 144 h of post-exposure) was the within-subject factor, the treatment (control, 0.23, 0.62, and 1.5 mg N-NH_3_/L) was the between-subjects factor, and the time (in seconds) spent by the first, second, and third animals to reach the food, the percentage of animals eating, and the mean distance to the food (in mm) were the response variables

Source of variation	Degrees of freedom	F	*p*	Effect size
*Time taken by the first animal to reach the food (t1)*				
Within-subject				
Time	2/56	54.8	< 0.00001	0.54
Time ✕ Treatment	6/56	4.1	< 0.01	0.21
Between subjects				
Treatment	3/28	7.5	< 0.001	0.24
*Time taken by the second animal to reach the food (t2)*				
Within-subject				
Time	2/56	44	< 0.00001	0.49
Time ✕ Treatment	6/56	1.5	0.2	0.09
Between subjects				
Treatment	3/28	6.1	< 0.01	0.2
*Time taken by the third animal to reach the food (t3)*				
Within-subject				
time	3/84	60.3	< 0.00001	0.53
Time ✕ Treatment	9/84	2	0.07	0.1
Between subjects				
Treatment	3/28	6.9	< 0.01	0.3
*Percentage of animals eating*				
Within-subject				
Time	2/56	30.6	< 0.00001	0.42
Time ✕ Treatment	6/56	4.4	< 0.01	0.24
Between subjects				
Treatment	3/28	4.5	< 0.05	0.14
*Mean distance to the food*				
Within-subject				
Time	2/56	73.7	< 0.00001	0.6
Time ✕ Treatment	6/56	8.2	< 0.00001	0.34
Between subjects				
Treatment	3/28	8.8	< 0.001	0.29

## Discussion

Our study has shown that some of the feeding behavioral variables (i.e., the time taken by the third animal to reach the food and the distance of animals to the food) were more sensitive than movement variables (i.e., percentage of immobile animals and the time taken by each animal to start the sliding movement without food). Our results also showed that, in general, the recovery period was efficient to recover the behavior of *P. antipodarum* after unionized ammonia exposure. Our feeding behavioral variables were integrative as they include all the aspects that animals need to reach food (i.e., movements, the perception of chemical stimuli from food, etc.). Two of these variables (mean distance to the food and time taken to reach the food by the third animal) were more sensitive to unionized ammonia (NOEC of 0.23 mg N-NH_3_/L and LOEC of 0.23 mg N-NH_3_/L, respectively) than movements in absence of food (NOEC of 0.62 mg N-NH_3_/L).

Unionized ammonia is a toxicant that cause deleterious effects on survival and feeding of several aquatic species. For instance, this toxicant reduced the egestion rate of the freshwater amphipod *Eulimnogammarus toletanus* after 6 days of continuous exposure at 0.30 mg N-NH_3_/L (Alonso and Camargo 2004). For a 2-days exposure, this species showed a NOEC of 0.30 mg N-NH_3_/L and a LC50 of 0.80 mg N-NH_3_/L (Alonso and Camargo 2004). In our study, the lowest NOEC value for the same exposure time was less than 0.23 mg N-NH_3_/L for mean distance to the food. Unionized ammonia also caused a cessation in feeding in the marine fish *Rachycentron canadum* at 96 h of exposure, with an EC50 of 0.62 mg N-NH_3_/L (Rodrigues et al. 2007), feeding behavior being more sensitive than swimming (EC50 = 0.80 mg N-NH_3_/L) and mortality (LC50 = 1.13 mg N-NH_3_/L) for this species. The freshwater planarian *Polycelis felina* showed a high long-term sensitivity to unionized ammonia*,* with a LOEC value at 30 days for mortality of 0.05 mg N-NH_3_/L and 0.02 mg N-NH_3_/L for movement, and an EC50 at 48 h for immobility of 0.33 mg N-NH_3_/L and 0.47 mg N-NH_3_/L for mortality (Alonso and Camargo 2006, 2011). Regarding the freshwater gastropods *Pleurocera unciale* and *Bellamya aeuruginosa*, unionized ammonia caused adverse effects (LC50 at 96 h) at concentrations of 0.61 mg N-NH_3_/L (Goudreau et al. 1993) and 0.56 mg N-NH_3_/L (Liu et al. 2021), respectively, which are higher than our lowest LOEC (0.23 mg N-NH_3_/L). In general, our study shows that mean distance to the food is a sensitive parameter, as its NOEC (< 0.23 mg N-NH_3_/L at 48 h of exposure) was relatively low. This concentration was higher than the safe concentrations of unionized ammonia at long-term exposures (ranged from 0.01 to 0.10 mg N-NH_3_/L) (Alonso and Camargo 2011). However, our NOEC was relatively low for a short-term exposure (48 h) in comparison with water long-term criteria. This may indicate that the mean distance to the food could be a suited variable to assess the effects of the exposure to the unionized ammonia.

Unionized ammonia may trigger several impairments that may help to explain the behavioral effects observed in our study. For instance, this toxicant presents a high solubility in lipids that, together with the absence of charge, cause a rapid absorption through cell membranes (Fromm and Gillette 1968). Therefore, during the first hours of exposure animals may uptake a high amount of unionized ammonia. Subsequently, this compound acts on several physiological aspects: causing damages to gills, affecting hemolymph pH, altering metabolism of muscle elements, damaging DNA, reducing viability of cells, or affecting neurotransmission, among others (Armstrong et al. 1978; Cong et al. 2017; Fromm and Gillette 1968; Zhang et al. 2020). These effects may get worse if external concentration of ammonia keeps high, as gills are involved in the excretion of ammonia (Maltby 1995). These adverse effects may help to explain the deleterious effects on animal movements and feeding behaviors that were observed in our study, as a depletion in oxygen uptake, neurotransmission alteration, and muscle degradation are main factors controlling movement capacity and feeding (Alonso and Camargo 2011; Cong et al. 2017).

Adverse effects of unionized ammonia in the perception of chemical stimuli by aquatic invertebrates have been previously reported (Edwards et al. 2018). In this sense, the perception of chemical stimuli released from food is a key element in food searching by animals (Azizishirazi et al. 2013; Hassenklöver et al. 2012; Lahman and Moore 2015; Kamio and Derby 2017). Toxicants may alter this mechanism directly through the impartment of chemosensory systems and/or by alterations of the physiological status, which could hinder the processing of information (Edwards et al. 2018; Sutterlin 1974). Toxicants interfere indirectly with chemical stimuli by masking the background signal (Edwards et al. 2018). This is an immediate mechanism, as no previous physiological impairment in the animals is necessary. This fact could contribute to explain the higher sensitivity of feeding behaviors over movement without food in our study. In fact, tracking odors from their sources (e.g., food) is one of the main factors involved in the movement of aquatic gastropods, and other animals (Kamio and Derby 2017; Wyeth 2019). In our study, the use of still water means that chemotaxis (i.e., the movement of snails in the direction of increasing chemical gradient) could be the main mechanisms of *P. antipodarum* to detect the food (Wyeth 2019). On the other hand, the absence of food in bioassays without toxicants usually causes an increase in animal activity (Alonso 2021). However, this trend was not observed in our study, which could indicate an adverse effect of unionized ammonia.

In general, behavioral bioassays have shown some advantages in ecotoxicology. They are less time-consuming than bioassays based on development and reproduction, and they have higher sensitivity than lethal bioassays (Melvin and Wilson 2013). Among the behavioral endpoints studied, our results have shown that those based on feeding behaviors are able to detect adverse effects of toxicants, during and after toxicant exposure. This is an agreement with a previous study in our laboratory with acetone (Alonso et al. 2016). Therefore, feeding behavior (i.e., all kind of behavioral activities that allow animals getting food) may be a promising candidate for the development of standardized protocols in ecotoxicological risk assessment based on behavior.

## Conclusions

We conclude that some of the behaviors monitored in the presence of food were more sensitive than movement variables monitored without food. Both groups of behaviors showed a higher sensitivity than mortality. Additionally, animals after unionized ammonia exposure showed a recovery of most of the behavioral endpoints. The inclusion of post-exposure periods and feeding behavior in ecotoxicological bioassays may be a relevant improvement in the realism of those studies, which is crucial to a proper ecotoxicological risk assessment.

## Supplementary Information

Below is the link to the electronic supplementary material.

Supplementary file1 (DOCX 21 KB)

Supplementary file2 (XLSX 19 KB)

Supplementary file3 (XLSX 20 KB)

## Data Availability

Raw data that support the findings of this study are available on request from the corresponding author once the paper is accepted for publication.

## Code Availability

The R code that supports the findings of this study is available on request from the corresponding author once the paper is accepted for publication.
