# Supplementary material for: Behavioral Variables to Assess the Toxicity of Unionized Ammonia in Aquatic Snails: Integrating Movement and Feeding Parameters
Source: Arch Environ Contam Toxicol. 2022 Mar 24;82(3):429–38. doi: 10.1007/s00244-022-00920-z (PMC8971178; doi:10.1007/s00244-022-00920-z)
Supplement: Supplementary file 1 — Supplementary file1 (DOCX 21 KB) [file 244_2022_920_MOESM1_ESM.docx]

**SUPPLEMENTARY MATERIAL S1**

This document describes the raw data collected during experiments. They are supplied in two Excel files:

- “Activity.xlsx”: this file contains the data related to the activity, immobility and mortality of snails during the experimental period. The column “time_exp” includes the time when the variables were monitored (at 48 h of exposure and 48 and 144 h of post-exposure); the “treat” and “rep” columns indicate the treatment (control (0) and the three unionized ammonia concentrations in mg N-NH_3_/L (1, 2 and 3)) and the 8 replicates per treatment respectively; the “activity” column indicates the mean reaction time (in seconds) of the six individuals of each replicate; the “immov” and “mort” columns are the mean proportion of immobile individuals and the mean of the cumulative proportion of dead individuals in each replicate.
- “Feeding.xlsx”: this file includes the data concerning the time taken by the first, second, and third animal to reach the food, animals eating and distance to the food. The column “time_exp” includes the time when the variables were monitored (at 48 h of exposure and 48 and 144 h of post-exposure); the “treat” and “rep” columns indicate the treatment (control (0) and the three unionized ammonia concentrations in mg N-NH_3_/L (1, 2 and 3)) and the 8 replicates per treatment respectively; “t1”, “t2” and “t3” are the time (in seconds) taken by the first, second and third animals, respectively, in each replicate to reach the food pellets; the “eating” column represents the percentage of animals that were observed eating in each replicate; the “distance” column is the mean distance (in mm) to the food in each replicate.
